# Safety and effectiveness of eribulin in Japanese patients with locally advanced or metastatic breast cancer: a post-marketing observational study

**DOI:** 10.1007/s10637-017-0486-4

**Published:** 2017-06-29

**Authors:** Junichiro Watanabe, Yoshinori Ito, Shozo Ohsumi, Mitsuhiro Mizutani, Hideya Tashiro, Kenichi Sakurai, Masato Takahashi, Tsuyoshi Saito, Junji Tsurutani, Hirofumi Mukai, Tetsuhiro Yoshinami, Shintaro Takao, Yasuhisa Yamamoto, Toshiyuki Matsuoka, Hirotaka Iwase, Hiroji Iwata, Seigo Nakamura, Toshiaki Saeki

**Affiliations:** 1 Division of Breast Oncology, Shizuoka Cancer Center, 1007 Shimonagakubo, Nagaizumi-cho, Sunto-gun, Shizuoka, 411-8777 Japan; 2 Department of Breast Medical Oncology, The Cancer Institute Hospital of Japanese Foundation for Cancer Research, 3-8-31, Ariake, Koto-ku, Tokyo 135-8550 Japan; 3 Department of Breast Oncology, National Hospital Organization, Shikoku Cancer Center, 160, Minamiumemotomachikou, Matsuyamashi, Ehime 791-0280 Japan; 4 Mikawa Breast Cancer Clinic, 39-6, Koita, Sasame-cho, Anjo, Aichi 446-0073 Japan; 5 Department of Surgery, Oita Prefectural Hospital, 476, Bunyou, Oita, Oita 870-8511 Japan; 6 Department of Breast Surgery, Nihon University School of Medicine, 30-1, Ohyaguchi Kami-cho, Itabashi-ku, Tokyo 173-8610 Japan; 7 Department of Breast Surgery, National Hospital Organization, Hokkaido Cancer Center, 2-3-54, Kikusuishijo, Shiroishi-ku, Sapporo, Hokkaido 003-0804 Japan; 8 Breast Surgery Unit, Japanese Red Cross Saitama Hospital, 1-5, Shintoshin, Chuo-ku, Saitama, Saitama 330-8553 Japan; 9 Department of Medical Oncology, Kinki University Faculty of Medicine, 377-2, Onohigashi, Osaka-sayama, Osaka 589-8511 Japan; 10 Division of Breast and Medical Oncology, National Cancer Center Hospital East, 6-5-1, Kashiwanoha, Kashiwa, Chiba 277-8577 Japan; 11 Department of Clinical Oncology, Osaka Medical Center for Cancer and Cardiovascular Diseases, 1-3-3, Nakamichi, Higashinari-ku, Osaka, Osaka 537-8511 Japan; 12 Department of Breast Surgery, Hyogo Cancer Center, 13-70, Kitaojicho, Akashi, Hyogo 673-8558 Japan; 13 Department of Surgery, Oomoto Hospital, 1-1-5, Oomoto, Kita-ku, Okayama, Okayama 700-0924 Japan; 14 Oncology PMS Section, Oncology Medical Department, Medical Division, Eisai Co., Ltd., 4-6-10 Koishikawa, Bunkyo-ku, Tokyo 112-8088 Japan; 15 Department of Breast and Endocrine Surgery, Graduate School of Medical Sciences, Kumamoto University, 1-1-1 Honjo, Chuo-ku, Kumamoto, Kumamoto 860-8556 Japan; 16 Division of Immunology, Aichi Cancer Center Research Institute, 1-1 Kanokoden, Chikusa-ku Nagoya, Aichi 464-8681 Japan; 17 Division of Breast Surgical Oncology, Department of Surgery, Showa University, 1-5-8 Hatanodai, Shinagawa-ku, Tokyo 142-8555 Japan; 18 Department of Breast Oncology, Saitama Medical University International Medical Center, 1397-1, Yamane, Hidaka, Saitama 350-1298 Japan

**Keywords:** Breast cancer, Eribulin, Japan, Post-marketing surveillance, Real world

## Abstract

*Background* This large-scale study was conducted to evaluate the safety and effectiveness of eribulin for the treatment of inoperable or recurrent breast cancer in real-world settings in Japan. *Methods* Between July and December 2011, eligible patients with inoperable or recurrent breast cancer receiving eribulin for the first time were centrally registered and observed for 1 year. Eribulin was administered intravenously (1.4 mg/m^2^) on days 1 and 8 of every 3-week cycle. The primary endpoint was the frequency and intensity of adverse drug reactions (ADRs). Secondary endpoints included overall response rate (ORR) and time to treatment failure (TTF). *Results* Of 968 patients registered at 325 institutions, 951 and 671 were included in the safety and effectiveness analyses, respectively. In the safety population, ADRs were observed in 841 patients (88.4%). The most common (≥15% incidence) were neutropenia (66.6%), leukopenia (62.4%), lymphopenia (18.4%), and peripheral neuropathy (16.8%). The most common grade ≥ 3 ADRs (>5% incidence) were neutropenia (59.8%), leukopenia (50.5%), lymphopenia (16.1%), and febrile neutropenia (7.7%). In the effectiveness population, ORR was 16.5% (95% confidence interval: 13.7, 19.4). The median TTF was 127 days (95% confidence interval: 120, 134). *Conclusions* The safety and effectiveness profile of eribulin was consistent with prior studies. Eribulin had a favorable risk-benefit balance when used in real-world clinical settings.

**Electronic supplementary material:**

The online version of this article (doi:10.1007/s10637-017-0486-4) contains supplementary material, which is available to authorized users.

## Introduction

Breast cancer is the second most common cancer in the world and the most common cancer among women, with 1.67 million new cases diagnosed in 2012 [1]. In Japan, breast cancer was the sixth leading cause of death among women in 2014 (20.6/100,000) [2]. Despite improvements in treatment, metastatic breast cancer (MBC) remains incurable and is the most common cause of death among patients with breast cancer [3, 4]; therefore, the goals of therapy are to prolong survival, palliate symptoms, and improve quality of life [5].

Anthracycline- and taxane-based regimens are currently the standard of care for adjuvant and first-line treatment for MBC. However, the long-term survival of patients with MBC remains poor. The 5-year survival of patients with stage IV breast cancer can be as low as 21%, in comparison to 100% in patients with stage I breast cancer [6]. In Japan, 5- and 10-year relative survival rates for patients with stage IV breast cancer were as low as 32.6% and 15.6%, respectively [7]. In addition, few options are available for treatment of patients with MBC who have been pre-treated with anthracyclines and taxanes, or those who have become resistant to anthracyclines and taxanes [3]. Thus, alternative treatment options that provide survival benefits for MBC patients are warranted.

Eribulin, a synthetic derivative of halichondrin B isolated from *Halichondria okadai*, is a new non-taxane microtubule dynamics inhibitor with a mechanism of action distinct from currently available taxanes. Unlike taxanes, eribulin binds to a single site on tubulin and to a small number of sites at microtubule ends [8]. Owing to its unique mechanism of action, eribulin displays antitumor activity in patients with well-defined taxane resistance [9].

Eribulin has received approval from the United States Food and Drug Administration and the European Medicines Agency for the treatment of locally advanced breast cancer and MBC refractory to both anthracyclines and taxanes [10]. EMBRACE, a randomized, phase III study in patients with heavily pre-treated MBC, reported a significant and clinically meaningful improvement in overall survival (OS) in patients treated with eribulin (median OS, 13.1 months; 95% confidence interval [CI]: 11.8, 14.3) compared with those who received physician’s choice of treatment (median OS, 10.6 months; 95% CI: 9.3, 12.5; hazard ratio [HR], 0.81; 95% CI: 0.66, 0.99; *p* = 0.041) [11]. In another randomized, phase III study that included pre-treated patients with locally advanced breast cancer or MBC, median OS in the eribulin group was 15.9 months, compared with 14.5 months in the capecitabine group (HR, 0.88; 95% CI: 0.77 1.00; *p* = 0.056) [12]. A pooled analysis of these two phase III studies demonstrated that eribulin significantly prolonged OS compared to the control (median OS, 15.2 months vs 12.8 months; HR, 0.85; 95% CI: 0.77, 0.95; *p* = 0.003); OS data also favored eribulin in the various subgroups assessed [13]. Since toxicity does not increase during long-term treatment, eribulin can help maintain stable disease while providing high quality of life [14]. In addition, phase II studies have demonstrated the antitumor activity of eribulin with a manageable tolerability profile in extensively pre-treated patients who had previously received an anthracycline, taxane, and capecitabine [15–17].

In Japan, eribulin was approved for the treatment of inoperable or recurrent breast cancer in April 2011, following its approval in the United States (November 2010), Singapore (February 2011), and Europe (March 2011). However, the phase II eribulin study conducted in Japan included only 81 patients, and pre-marketing clinical studies included only a small number of Japanese patients [15]. Given the limited evidence of eribulin’s safety and effectiveness, specifically in Japanese populations, our current observational study was conducted as a post-marketing commitment to the Ministry of Health, Labour and Welfare of Japan to assess the safety and effectiveness of eribulin in patients with inoperable or recurrent breast cancer in routine clinical settings in Japan.

## Methods

### Study design

This was a post-marketing, observational study conducted in 325 centers to evaluate the safety and effectiveness of eribulin mesylate (Halaven®, Eisai Co., Ltd., Japan) in Japanese patients with inoperable or recurrent breast cancer (ClinicalTrials.gov ID:NCT01463891). Patients were enrolled from July 19, 2011 (the launch date of Halaven®), to December 17, 2011, and were observed for 1 year following enrollment. Patients who discontinued treatment within 1 year were observed until the end of the cycle in which treatment was discontinued. Patients who continued treatment with eribulin for more than 1 year were observed through the treatment cycle ending at the 1 year mark. The study was conducted in accordance with the Declaration of Helsinki and Japanese regulatory requirements stipulated in Good Post-Marketing Study Practices (GPSP). Approval from the institutional ethics committee/institutional review board was obtained prior to commencement of the study. For this type of study formal consent was not required.

### Patients

Patients with inoperable or recurrent breast cancer receiving treatment with eribulin for the first time were registered in this study by central registration. At institutions with study contracts specifying the number of patients to be registered, patients receiving their first treatment with eribulin were enrolled until this target number was reached. Patients with contraindications to treatment (high myelosuppression, known hypersensitivity to eribulin, pregnancy, or the possibility of pregnancy) were excluded [18].

### Treatment

Eribulin was administered intravenously at a dose of 1.4 mg/m^2^ on days 1 and 8 of every 3-week cycle. Dosing was adjusted or discontinued depending on the condition of individual patients.

### Assessments

The primary endpoint was the frequency and intensity of adverse drug reactions (ADRs). Safety was assessed throughout the study by recording adverse events (AEs). Severity and causality in relation to eribulin were assessed for each AE; when a causal relationship could not be ruled out, the AE was considered an ADR. ADRs that were not consistent with eribulin’s prescribing information were considered unexpected. AEs and ADRs were graded according to the Japanese version of Common Terminology Criteria for Adverse Events (version 3.0) and tabulated using the Japanese version of the Medical Dictionary for Regulatory Activities (version 16.1).

Patients with hepatic function disorder were defined as those with aspartate aminotransferase (AST) or alanine aminotransferase (ALT) levels >2.5 times the upper limit of normal (ULN), or total bilirubin (T-Bil) levels >1.5 times the ULN, before the start of eribulin. Patients whose pre-treatment levels of AST, ALT, and T-Bil were unavailable were designated as having unknown hepatic function status. Patients with renal function disorder were defined as those with serum creatinine (SCr) levels >1.5 times the ULN before the start of eribulin. Patients whose pre-treatment SCr data were unavailable were classified as patients with unknown renal function status.

Secondary endpoints included overall response rate (ORR) and time to treatment failure (TTF). Effectiveness was evaluated using best overall response, as determined by individual physicians at each study center according to Response Evaluation Criteria in Solid Tumors version 1.1. Imaging techniques included computed tomography, magnetic resonance imaging, and X-rays. These evaluations were scheduled according to the clinical practice standards of each institution rather than by a protocol of fixed intervals. Response was classified as follows: complete response (CR), partial response (PR), stable disease (SD), progressive disease (PD), and not evaluable (NE). ORR was defined as CR + PR; disease control rate (DCR) was defined as CR + PR + SD; and clinical benefit rate (CBR) was defined as CR + PR + SD ≥6 months. TTF was defined as the time from the first dose of eribulin until the date of treatment discontinuation from any cause (e.g., death, documentation of disease progression, adverse events, or patient’s request), or was censored at the date of last follow-up for surviving patients remaining on treatment.

### Statistical analysis

A sample size of 500 patients was estimated to be large enough to detect at least one case of severe infection (known frequency 0.5%) at a probability of 90%. Fisher’s exact test was used for comparison between 2 groups, whereas a chi-square test was used for comparison among 3 or more groups. The Kaplan-Meier method was used to calculate TTF. All analyses were performed using Statistical Analysis System (SAS) Version 9.1.3 (SAS Institute Inc., Cary, NC, USA).

## Results

### Patient disposition and baseline characteristics

A total of 968 patients were registered at 325 institutions. The safety analysis included 951 patients; 17 were excluded due to a history of eribulin administration, refusal to fill in the case report forms, and lack of eribulin administration after registration. Of these, 671 patients were included in the effectiveness analysis; 280 were excluded due to a lack of diagnostic imaging data.

Baseline characteristics were similar between the safety and effectiveness populations (Table 1). In the safety population, median age of patients was 59.0 years (range, 26–88). The proportion of patients with human epidermal growth factor receptor type 2 (HER2)-positive status was 18.4% in the safety population and 18.5% in the effectiveness population; estrogen receptor (ER)-positive, 67.4% and 70.3%; progesterone receptor (PgR)-positive, 49.6% and 51.7%; and triple negative, 18.4% and 16.4%. The median number of previous chemotherapy regimens was 4.0 (range, 0–14).

**Table 1:** Patient demographics and baseline characteristics

	Safety analysis set *n* = 951		Effectiveness analysis set *n* = 671	
	*n*	(%)	*n*	(%)
Gender				
Female	949	(99.8)	669	(99.7)
Male	2	(0.2)	2	(0.3)
Age (years)				
≤ 64	701	(73.7)	499	(74.4)
65–74	204	(21.5)	143	(21.3)
≥ 75	46	(4.8)	29	(4.3)
Median (range)	59.0 (26–88)		59.0 (26–88)	
ECOG performance status				
0	481	(50.6)	362	(53.9)
1	364	(38.3)	251	(37.4)
2	86	(9.0)	51	(7.6)
3	18	(1.9)	6	(0.9)
4	2	(0.2)	1	(0.1)
HER2/neu				
Negative	703	(73.9)	495	(73.8)
Positive	175	(18.4)	124	(18.5)
Unknown	73	(7.7)	52	(7.7)
ER				
Negative	285	(30.0)	186	(27.7)
Positive	641	(67.4)	472	(70.3)
Unknown	25	(2.6)	13	(1.9)
PgR				
Negative	444	(46.7)	304	(45.3)
Positive	472	(49.6)	347	(51.7)
Unknown	35	(3.7)	20	(3.0)
Triple negative				
No	745	(78.3)	542	(80.8)
Yes	175	(18.4)	110	(16.4)
Unknown	31	(3.3)	19	(2.8)
Metastatic lesions				
Breast	97	(10.2)	65	(9.7)
Lymph nodes	450	(47.3)	332	(49.5)
Lung	425	(44.7)	305	(45.5)
Liver	482	(50.7)	344	(51.3)
Bone	531	(55.8)	376	(56.0)
Brain	118	(12.4)	80	(11.9)
Skin	155	(16.3)	97	(14.5)
Others	165	(17.4)	120	(17.9)
Hepatic dysfunction^a^				
No	803	(84.4)	580	(86.4)
Yes	106	(11.1)	63	(9.4)
Unknown	42	(4.4)	28	(4.2)
Renal impairment^b^				
No	859	(90.3)	610	(90.9)
Yes	12	(1.3)	8	(1.2)
Unknown	80	(8.4)	53	(7.9)
Number of previous chemotherapy regimens^c^				
0	41	(4.3)	28	(4.2)
1	68	(7.2)	51	(7.6)
2	149	(15.7)	108	(16.1)
3	161	(16.9)	116	(17.3)
4	155	(16.3)	109	(16.2)
≥ 5	377	(39.6)	259	(38.6)
Median (range)	4.0 (0–14)		4.0 (0–14)	

*ECOG* Eastern Cooperative Oncology Group, *ER* estrogen receptor, *HER2/neu* human epidermal growth factor receptor type2, *PgR* progesterone receptor

^a^Patients with hepatic function disorder were defined as those showing aspartate aminotransferase (AST) or alanine aminotransferase (ALT) levels of >2.5 times the upper limit of normal (ULN) or total bilirubin (T-Bil) levels of >1.5 times the ULN before the start of eribulin. Patients whose pre-treatment data were unavailable for AST, ALT, and T-Bil were counted as patients with unknown hepatic function status

^b^Patients with renal function disorder were defined as those showing serum creatinine (SCr) levels of >1.5 times the ULN before the start of eribulin. Patients whose pre-treatment SCr data were unavailable were counted as patients with unknown renal function status

^c^Chemotherapy for inoperable or recurrent breast cancer

### Dose exposure

In the safety population, 71.7% of patients received an initial eribulin dose of 1.4 mg/m^2^, 19.1% received 1.1 mg/m^2^, and 3.8% received 0.7 mg/m^2^. Treatment lasted for a median of 4 cycles (range, 1–19), and median duration of exposure to eribulin was 14.1 weeks (range, 3–59). Median relative dose intensity was 0.75 (range, 0.21–1.25). Eribulin was administered concomitantly with chemotherapy, hormone therapy, or radiotherapy in 7.7%, 16.3%, and 5.8% of patients, respectively (Table 2). Trastuzumab was used in combination with eribulin in HER2-positive patients (34.3%). Dose exposure was comparable in the effectiveness population.

**Table 2:** Dose exposure to eribulin

	Safety analysis set *n* = 951		Effectiveness analysis set *n* = 671	
	*n*	(%)	*n*	(%)
Initial dose (mg/m^2^)				
1.4	682	(71.7)	489	(72.9)
1.1	182	(19.1)	121	(18.0)
0.7	36	(3.8)	24	(3.6)
Other^a^	51	(5.4)	37	(5.5)
Number of cycles				
Median (range)	4.0 (1–19)		5.0 (1–19)	
Duration of exposure (weeks)				
Median (range)	14.1 (3–59)		18.0 (3–59)	
Number of administrations (times)				
Median (range)	8.0 (1–36)		10.0 (1–36)	
Relative dose intensity				
Median (range)	0.750 (0.21–1.25)		0.750 (0.21–1.03)	
Concomitant chemotherapy				
No	877	(92.2)	615	(91.7)
Yes	73	(7.7)	56	(8.3)
Unknown	1	(0.1)	0	-
Concomitant hormone therapy				
No	796	(83.7)	544	(81.1)
Yes	155	(16.3)	127	(18.9)
Concomitant radiotherapy				
No	896	(94.2)	629	(93.7)
Yes	55	(5.8)	42	(6.3)

^a^Initial dose of 0.8, 0.9, 1.0, 1.1, 1.2, or 1.3 mg/m^2^

### Safety analysis

A total of 841 patients (88.4%) reported ADRs. The most common (>10% incidence) ADRs observed were neutropenia (66.6%), leukopenia (62.4%), lymphopenia (18.4%), peripheral neuropathy (16.8%), alopecia (12.1%), nausea (11.3%), stomatitis (10.9%), and pyrexia (10.3%) (Table 3). Grade ≥ 3 ADRs with >5% incidence were neutropenia (59.8%), leukopenia (50.5%), lymphopenia (16.1%), and febrile neutropenia (7.7%). ADRs leading to death were reported in 6 patients (0.6%); these included pneumonia (0.2%), liver metastasis (0.1%), interstitial lung disease (0.1%), and pulmonary bleeding (0.1%), as well as sepsis (0.1%), tumor lysis syndrome (0.1%), and disseminated intravascular coagulation (0.1%). Sepsis, tumor lysis syndrome, and disseminated intravascular coagulation developed in the same patient. ADRs leading to discontinuation of treatment with eribulin were reported in 93 patients (9.8%); these included neutropenia (2.3%), leukopenia (2.3%), peripheral neuropathy (1.7%), febrile neuropathy (0.8%), lymphopenia (0.7%), anorexia (0.7%), thrombocytopenia (0.6%), malaise (0.6%), and interstitial lung disease (0.5%).

**Table 3:** Adverse drug reactions with an incidence higher than 5% (*n* = 951)

	All grade		≥Grade 3	
	*n*	(%)	*N*	(%)
Overall	841	(88.4)	665	(69.9)
Hematologic events				
Neutropenia	633	(66.6)	569	(59.8)
Leukopenia	593	(62.4)	480	(50.5)
Lymphopenia	175	(18.4)	153	(16.1)
Febrile neutropenia	73	(7.7)	73	(7.7)
Anemia	63	(6.6)	35	(3.7)
Non-hematologic events				
Peripheral neuropathy	160	(16.8)	26	(2.7)
Alopecia	115	(12.1)		N/A
Nausea	107	(11.3)	4	(0.4)
Stomatitis	104	(10.9)	16	(1.7)
Pyrexia	98	(10.3)	2	(0.2)
Malaise	93	(9.8)	7	(0.7)
Decreased appetite	81	(8.5)	10	(1.1)
AST increased	76	(8.0)	15	(1.6)
Dysgeusia	59	(6.2)	0	
ALT increased	52	(5.5)	15	(1.6)
C-reactive protein increased	51	(5.4)	6	(0.6)

*AST* aspartate aminotransferase, *ALT* alanine aminotransferase, *N/A* not available

### Effectiveness analysis

**Fig. 1:**
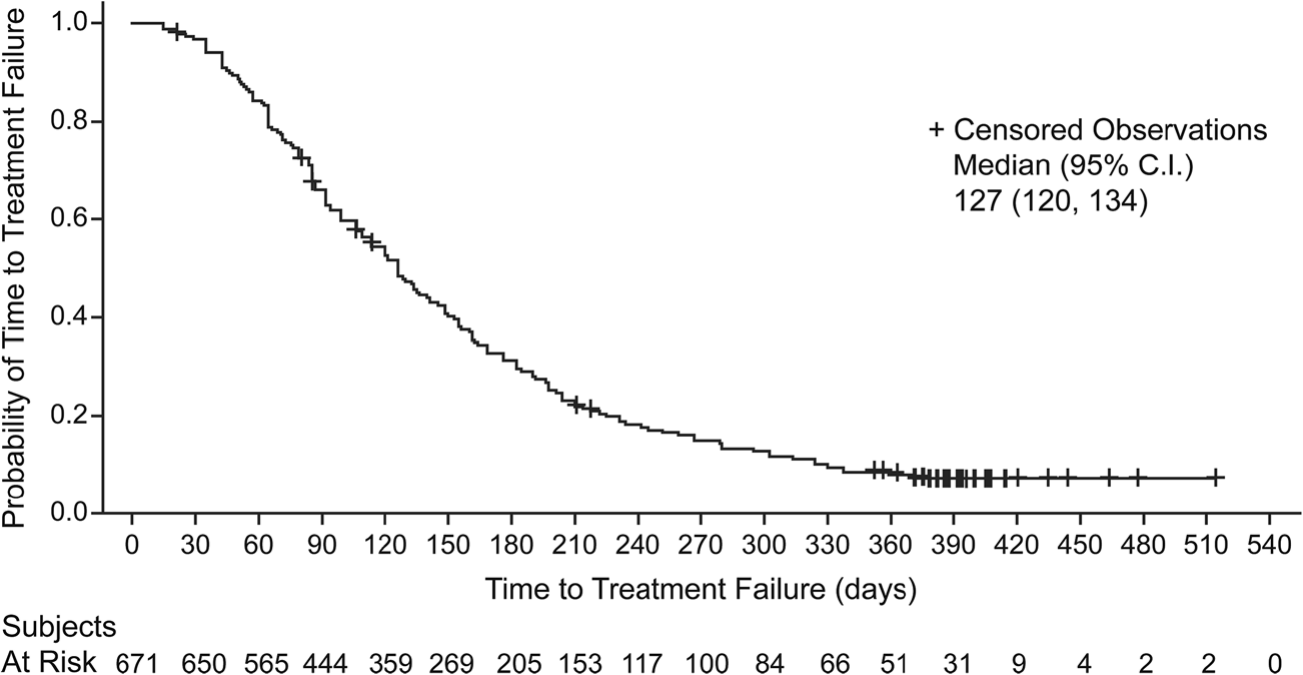
Kaplan-Meier analysis of time to treatment failure in the effectiveness population. *C.I.* confidence interval

CR and PR were observed in 1.3% and 15.2% of patients, respectively. The ORR was 16.5%, DCR was 50.1%, and CBR was 22.4% (Table 4). The median TTF was 127 days (95% CI: 120, 134), with 7.9% censored cases (Fig. 1). The primary reasons for treatment discontinuation (*n* = 618) were disease progression (86.1%), adverse events (6.6%), patient’s request (3.7%), and death (1.9%).

**Table 4:** Effectiveness analysis (*n* = 671)

	*n*	(%)
Best overall response		
Complete response	9	(1.3)
Partial response	102	(15.2)
Stable disease	225	(33.5)
Progressive disease	330	(49.2)
Not evaluable	5	(0.7)
Overall response rate (%)	111	(16.5)
95% CI (%)	(13.7, 19.4)	
Disease control rate (%)	336	(50.1)
95% CI (%)	(46.3, 53.9)	
Clinical benefit rate (%)	150	(22.4)
95% CI (%)	(19.2, 25.5)	

*CI* confidence interval

### Subanalysis

#### Dose exposure

A subanalysis of initial eribulin dose by age group revealed that the proportion of patients receiving an initial dose of 1.4 mg/m^2^ was smaller in patients aged ≥75 years (58.7%) than in patients aged ≤64 years (72.2%) or 65–74 years (73.0%). In addition, 52.8% of patients with hepatic dysfunction received an initial dose of 1.4 mg/m^2^, while 74.2% of patients without hepatic dysfunction received this initial dose (Online Resource 1).

#### Safety analysis

The incidence of ADRs of all grades in patients aged ≤64 years, 65–74 years, and ≥75 years was 87.7%, 91.2%, and 87.0%, respectively. Grade ≥ 3 ADRs in age groups ≤64 years, 65–74 years, and ≥75 years occurred at an incidence of 70.2%, 68.1%, and 73.9%, respectively (Online Resource 2). Subanalysis of safety by hepatic function revealed that the incidence of ADRs of all grades was significantly higher (*p* = 0.0369) in patients with hepatic dysfunction (94.3%) than in patients without hepatic dysfunction (87.4%). Compared to patients without hepatic dysfunction, patients with hepatic dysfunction reported a higher incidence of thrombocytopenia (15.1% vs 2.1%), febrile neutropenia (23.6% vs 5.7%), and stomatitis (18.9% vs 9.3%) (Table 5).

**Table 5:** Subanalysis of common (≥10% incidence) adverse drug reactions by hepatic function

	Without hepatic dysfunction *n* = 803				With hepatic dysfunction *n* = 106			
	All grades		≥Grade 3		All grades		≥Grade 3	
	*n*	(%)	*n*	(%)	*n*	(%)	*n*	(%)
Overall	702	(87.4)	573	(71.4)	100	(94.3)	92	(86.8)
Hematologic events								
Neutropenia	533	(66.4)	476	(59.3)	70	(66.0)	67	(63.2)
Leukopenia	494	(61.5)	389	(48.4)	75	(70.8)	72	(67.9)
Lymphopenia	149	(18.6)	128	(15.9)	23	(21.7)	22	(20.8)
Thrombocytopenia	17	(2.1)	8	(1.0)	16	(15.1)	9	(8.5)
Non-hematologic events								
Peripheral neuropathy	136	(16.9)	20	(2.5)	12	(11.3)	3	(2.8)
Alopecia	98	(12.2)		N/A	9	(8.5)		N/A
Nausea	89	(11.1)	2	(0.2)	14	(13.2)	2	(1.9)
Malaise	81	(10.1)	6	(0.7)	5	(4.7)	0	
Pyrexia	79	(9.8)	2	(0.2)	12	(11.3)	0	
Stomatitis	75	(9.3)	9	(1.1)	20	(18.9)	6	(5.7)
Febrile neutropenia	46	(5.7)	46	(5.7)	25	(23.6)	25	(23.6)

*N/A* not available

#### Effectiveness analysis

The ORR of patients aged ≥75 years (34.5%) was significantly higher (*p* = 0.0290) than that of patients aged ≤64 years (15.8%) or 65–74 years (15.4%). The relatively small number of patients ≥75 years (*n* = 46) suggests a possible bias; however, it is encouraging that older age was not associated with a lower ORR. In addition, the ORR of patients with hepatic dysfunction (25.4%) was higher, although not significantly so (*p* = 0.0502), than that of patients without hepatic dysfunction (15.5%). Patients receiving concomitant hormone therapy showed a significantly higher ORR (*p* < 0.001) than that of patients without hormone therapy (29.1% vs 13.6%). ORR was significantly lower in patients who had previously received a higher number of chemotherapy regimens (0 regimens, 35.7% vs ≥5 regimens, 12.4%; *p* = 0.0069) (Table 6).

**Table 6:** Subanalysis of ORR by age, hepatic function, hormone therapy, and history of chemotherapy

	Total patients *n*	Patients achieving response *n*	ORR %
Age (years)			
≤64	499	79	15.8
65–74	143	22	15.4
≥75	29	10	34.5
Hepatic dysfunction			
No	580	90	15.5
Yes	63	16	25.4
Unknown	28	5	17.9
Concomitant hormone therapy			
No	544	74	13.6
Yes	127	37	29.1
History of chemotherapy			
0	28	10	35.7
1	51	14	27.5
2	108	15	13.9
3	116	20	17.2
4	109	20	18.3
≥5	259	32	12.4

*ORR* overall response rate

## Discussion

To our knowledge, this is the first large-scale, post-marketing observational study to examine the safety and effectiveness of eribulin in Japanese patients with inoperable or recurrent breast cancer in a real-world setting. Prior to this study, the only clinical research evaluating eribulin in Japanese patients with heavily pre-treated MBC was a phase II study of 81 patients [15]. By contrast, the current study included 951 patients and evaluated the safety and effectiveness of eribulin during its use in clinical practice.

The safety profile of eribulin in this study was largely consistent with the prior Japanese phase II study [15], with neutropenia, leukopenia, and lymphopenia as the most commonly reported ADRs. The current study reported ADRs rather than AEs as in the phase II study; however, there was a striking similarity between the most common ADRs and AEs in both studies. In the phase II study, the most frequently (≥50% incidence) occurring AEs included neutropenia (98.8%), leukopenia (98.8%), and lymphopenia (54.3%) [15], whereas the most common ADRs (≥15% incidence) in the current study were neutropenia (66.6%), leukopenia (62.4%), and lymphopenia (18.4%). These findings also match those of other phase II [16, 17] and phase III [12] studies showing hematological toxicities as the most common AE/ADR during treatment with eribulin, with neutropenia reported most frequently (50–65%). Of note, grade 3/4 neutropenia may be more pronounced in East Asian populations; the frequency of grade 3/4 neutropenia in global trials was 20–65% [11, 12, 16, 17], compared to 85–95% in East Asian studies [15, 19]. Finally, the proportion of patients that discontinued treatment due to ADRs or AEs was 9.8% in the current study and 7.4% in the phase II study [15]. Therefore, the safety results from the current study corroborate the results from not only the previous phase II study in Japan, but other prior studies as well [11, 12, 16, 17].

Comparison of the incidence of ADRs among the three age groups (≤64 years, 65–74 years, and ≥75 years) in this study revealed no significant differences among the groups. The proportion of patients receiving eribulin at an initial dose of 1.4 mg/m^2^ was 72.2%, 73.0%, and 58.7% in the three age groups, respectively, indicating that the initial dose was more frequently adjusted for patients aged ≥75 years but did not differ markedly between the ≤64 years age group and the 65–74 years age group (Online Resource 1). Taken together, these data suggest that reducing the initial dose from 1.4 mg/m^2^ to 1.1 mg/m^2^ or 0.7 mg/m^2^ for patients aged ≥75 years may help avoid ADRs.

In a previous study analyzing the pharmacokinetic parameters of eribulin in patients with hepatic function classified using the Child-Pugh system (normal, mild dysfunction [Child-Pugh A], and moderate dysfunction [Child-Pugh B]), both mild and moderate hepatic dysfunction was associated with reduction of clearance, extension of half-life, increase of area under the curve (after correction for dose level), and increase of peak serum concentration (after correction for dose level) of eribulin [20]. Based on these findings, eribulin dose reduction should be recommended for patients with impaired hepatic function. In the current study, the proportion of patients receiving eribulin at an initial dose of 1.4 mg/m^2^ was 21.4% lower in patients with hepatic dysfunction than in patients without hepatic dysfunction (Online Resource 1). Therefore, although the initial dose of eribulin was adjusted for hepatic function, the incidence of ADRs was still higher in patients with hepatic dysfunction (94.3%) than in patients without hepatic dysfunction (87.4%). This indicates the need for evaluating hepatic function markers sufficiently before the start of eribulin treatment and considering initial dose reductions for patients with hepatic dysfunction.

The effectiveness of eribulin was evaluated using best overall response, as determined by diagnostic imaging conducted during the entire treatment period. It is difficult to directly compare results between the current study and the prior Japanese phase II study due to differences in tumor assessment methods (investigator review in this study versus independent review in the phase II study). However, the ORR of 16.5% in the current study (95% CI 13.7, 19.4) did not differ significantly from the ORR of 21.3% in the phase II study (95% CI 12.9, 31.8). In addition, response rate in the current study was higher than that of previous phase II [16, 17] and phase III [12] studies (approximately 9–14%).

In the current study, the median TTF was 127 days. In prior phase II studies reported by Aogi et al. [15], Cortes et al. [16], and Vahdat et al. [17], median progression-free survival (PFS) was 3.7 months, 2.6 months, and 79 days, respectively. Median PFS in the eribulin group was 3.7 months in the randomized, phase III EMBRACE study [11]. In another randomized, phase III study [12], the median PFS in the eribulin group was 4.1 months. While caution is warranted when directly comparing TTF and PFS, TTF in the current study was longer than previously reported values of PFS from phase II [15–17] and phase III studies [11, 12]. In the current study, the interval between tumor assessments was not predetermined by protocol, but was instead performed according to the clinical practice standards of each institution; thus, TTF in the current study accurately reflects the utility of eribulin in the real-world setting.

Our analysis of factors affecting response to treatment indicated that ORR was higher in patients with hepatic dysfunction and patients receiving concomitant hormone therapy, and lower in patients that had previously received a large number of chemotherapy regimens for inoperable or recurrent breast cancer. As discussed previously, the initial dose of eribulin was reduced in patients with hepatic dysfunction; therefore, it is possible that the plasma concentration of eribulin was maintained at a steady state in these patients, resulting in higher effectiveness in patients with disturbed hepatic drug metabolism or excretion. Regarding the previous use of chemotherapy for inoperable or recurrent breast cancer, the prior Japanese phase II study also reported a decrease in ORR associated with an increase in the number of chemotherapy regimens (36.0% for 0–1 regimen, 14.7% for 2 regimens, and 14.3% for 3 regimens) comparable with ORRs reported in the current study (0 regimens, 35.7%, ≥5 regimens, 12.4%; *p* = 0.0069).

We identified two inherent limitations of this study. Of 951 patients included in this study (safety population), 671 patients were included in effectiveness analysis, and 280 patients were excluded due to a lack of diagnostic imaging data. Therefore, almost 30% of enrolled patients were not included in the effectiveness analysis. This can be interpreted as a reflection of real-world clinical practice where tumor response assessments may not be conducted routinely, and as a limitation of observational studies of chemotherapeutic agents. In addition, the study design and limited observation period (1 year) did not allow for sufficient survival analysis.

The results of this study showed safety and effectiveness profiles of eribulin similar to those seen in the pre-marketing clinical trial when used for patients with inoperable or recurrent breast cancer during clinical practice. Eribulin demonstrated a favorable risk-benefit balance when used in real-world clinical settings.

## Electronic supplementary material

Online Resource 1 (DOCX 30 kb)

Online Resource 2 (DOCX 50 kb)
