# Supplementary material for: Safety and effectiveness of eribulin in Japanese patients with locally advanced or metastatic breast cancer: a post-marketing observational study
Source: Invest New Drugs. 2017 Jun 29;35(6):791–9. doi: 10.1007/s10637-017-0486-4 (PMC5694520; doi:10.1007/s10637-017-0486-4)
Supplement: Supplementary file 1 — (DOCX 30 kb) [file 10637_2017_486_MOESM1_ESM.docx]

**Online Resource 1** Subanalysis of initial dose (1.4 mg/m^2^) by age and hepatic function

|  | Safety analysis set | | | Effectiveness analysis set | | |
| --- | --- | --- | --- | --- | --- | --- |
|  | Total | Initial dose of 1.4 mg/m^2^ | | Total | Initial dose of 1.4 mg/m^2^ | |
|  | *N* | *n* | (%) | *N* | *n* | (%) |
| Age (years) |  |  |  |  |  |  |
| ≤64 | 701 | 506 | (72.2) | 499 | 366 | (73.3) |
| 65–74 | 204 | 149 | (73.0) | 143 | 106 | (74.1) |
| ≥75 | 46 | 27 | (58.7) | 29 | 17 | (58.6) |
| Hepatic dysfunction | |  |  |  |  |  |
| No | 803 | 596 | (74.2) | 580 | 437 | (75.3) |
| Yes | 106 | 56 | (52.8) | 63 | 32 | (50.8) |
| Unknown | 42 | 30 | (71.4) | 28 | 20 | (71.4) |
