# Supplementary material for: Safety and effectiveness of eribulin in Japanese patients with locally advanced or metastatic breast cancer: a post-marketing observational study
Source: Invest New Drugs. 2017 Jun 29;35(6):791–9. doi: 10.1007/s10637-017-0486-4 (PMC5694520; doi:10.1007/s10637-017-0486-4)
Supplement: Supplementary file 2 — (DOCX 50 kb) [file 10637_2017_486_MOESM2_ESM.docx]

**Online Resource 2** Subanalysis of common (≥10% incidence) adverse drug reactions by age

|  | ≤64 years  *n* = 701 | | | | 65–74 years  *n* = 204 | | | | ≥75 years  *n* = 46 | | | |
| --- | --- | --- | --- | --- | --- | --- | --- | --- | --- | --- | --- | --- |
|  | All grades | | ≥Grade 3 | | All grades | | ≥Grade 3 | | All grades | | ≥Grade 3 | |
|  | *n* | (%) | *n* | (%) | *n* | (%) | *n* | (%) | *n* | (%) | *n* | (%) |
| Overall | 615 | (87.7) | 492 | (70.2) | 186 | (91.2) | 139 | (68.1) | 40 | (87.0) | 34 | (73.9) |
| Hematologic events |  |  |  |  |  |  |  |  |  |  |  |  |
| Neutropenia | 463 | (66.0) | 413 | (58.9) | 139 | (68.1) | 127 | (62.3) | 31 | (67.4) | 29 | (63.0) |
| Leukopenia | 437 | (62.3) | 350 | (49.9) | 127 | (62.3) | 107 | (52.5) | 29 | (63.0) | 23 | (50.0) |
| Lymphopenia | 128 | (18.3) | 110 | (15.7) | 38 | (18.6) | 34 | (16.7) | 9 | (19.6) | 9 | (19.6) |
| Non-hematologic events |  |  |  |  |  |  |  |  |  |  |  |  |
| Peripheral neuropathy | 113 | (16.1) | 14 | (2.0) | 39 | (19.1) | 9 | (4.4) | 8 | (17.4) | 3 | (6.5) |
| Alopecia | 86 | (12.3) |  | N/A | 23 | (11.3) |  | N/A | 6 | (13.0) |  | N/A |
| Nausea | 85 | (12.1) | 4 | (0.6) | 20 | (9.8) | 0 | (0.0) | 2 | (4.3) | 0 |  |
| Pyrexia | 81 | (11.6) | 2 | (0.3) | 15 | (7.4) | 0 | (0.0) | 2 | (4.3) | 0 |  |
| Malaise | 74 | (10.6) | 5 | (0.7) | 16 | (7.8) | 2 | (1.0) | 3 | (6.5) | 0 |  |
| Stomatitis | 67 | (9.6) | 9 | (1.3) | 29 | (14.2) | 5 | (2.5) | 8 | (17.4) | 2 | (4.3) |
| Decreased appetite | 53 | (7.6) | 5 | (0.7) | 22 | (10.8) | 3 | (1.5) | 6 | (13.0) | 2 | (4.3) |
| C-reactive protein increased | 37 | (5.3) | 4 | (0.6) | 9 | (4.4) | 0 |  | 5 | (10.9) | 2 | (4.3) |

*N/A* not available
